# Supplementary material for: Impacts of the Pandemic, Animal Source Food Retailers’ and Consumers’ Knowledge and Attitudes toward COVID-19, and Their Food Safety Practices in Chiang Mai, Thailand
Source: Int J Environ Res Public Health. 2022 Aug 17;19(16):10187. doi: 10.3390/ijerph191610187 (PMC9408671; doi:10.3390/ijerph191610187)
Supplement: Supplementary file 1 [file ijerph-19-10187-s001.zip › Supplementary Files/Supplementary File S1 QX_COVID-19-ASF Retailers.pdf]

Survey questionnaires [ID.01-ASF Retailers]: Assessing impacts of COVID-19 pandemic on animal source food retailers and consumers in Thailand (English translation)

A. Participant Information

A1. Type of ASF Retailer: *(select 1 appropriate answer according to the **MAIN** ASF type is sold)*

1. Pork 2. Poultry (chicken, duck, …) 3. Beef 4. Fish/Seafood

A2. Gender:

1. Male
2. Female

A3. Year of birth: …………… (e.g. 1980)

A4. Sub-district: ……………….……… **District:** ………………………

A5. Area:

1. Urban/Peri-urban
2. Rural

A6. Highest education level:

1. Illiterate
2. Elementary school
3. Secondary school
4. High school
5. Colleges, university, or higher degrees

A7. What is your business type? *(select 1 answer)*

1. Wholesale only
2. Retail only
3. Both retail and wholesale
4. Other (specify): …………………

B. Impact of COVID-19 to ASF Business and Income

B1. At each below period, what is <u>an average amount</u> (estimate) of ASF (Kg) you sell per day? *(fill the amount of ASF type (if any) which you have sold, or you are selling by each period)*

**ASF types** *(any applicable)*	**At the same time last year** *(before Feb/Mar 2020)*	**During partial lockdown** *(in weeks of April 2020)*	**At present** *(from May 2020 to now)*
Pork	…..	…..	…..
Poultry (chicken, duck, …)	…..	…..	…..
Beef	…..	…..	…..
Fish/seafood	…..	…..	…..

B2. At each below period, <u>from whom</u> do you often purchase animal source food type?
(select source type which applied) *(unit: Times/week), DK = don't know*

Market types, period, and frequency *(any applicable)*	At the same time last year *(before Feb/Mar 2020)*						During partial lockdown *(in weeks of April 2020)*						At present *(from May 2020 to now)*					
	Every day	3-6 times	1-2 times	< 1 time	Never	DK	Every day	3-6 times	1-2 times	< 1 time	Never	DK	Every day	3-6 times	1-2 times	< 1 time	Never	DK
Farmers																		
Wholesalers/ middleman																		
Slaughterhouse																		
Self-provide																		
Others (specify): ……..																		

B3. During partial lockdown *(in weeks of April 2020)*, how easy for you to buy animal source food for selling <u>COMPARED</u> to before COVID-19 outbreak? *(before Feb/Mar 2020, or at the same time last year)*

1. Easier
2. Normal
3. More difficult
4. Do not know

B4. At present *(from May 2020 to now)*, how easy for you to buy animal source food for selling <u>COMPARED</u> to before COVID-19 outbreak? *(before Feb/Mar 2020, or at the same time last year)*

1. Easier
2. Normal
3. More difficult
4. Do not know

B5. At each below period, <u>how many ASF shops</u> (like your meat type) in this market?

Period	Number of shops	Don't know
**Before COVID-19 outbreak** *(before Feb/Mar 2020, or at the same time last year)*	…..	…..
**During partial lockdown** *(in weeks of April 2020)*	…..	…..
**At present** *(from May 2020 to now)*	…..	…..

B6. At each below period, <u>which way and how frequent</u> have you operated the ASF selling? (times/week)

Selling way and frequency (any applicable)	At the same time last year *(before Feb/Mar 2020)*						During partial lockdown *(in weeks of April 2020)*						At present *(from May 2020 to now)*					
	Every day	3-6 times	1-2 times	< 1 time	Never	DK	Every day	3-6 times	1-2 times	< 1 time	Never	DK	Every day	3-6 times	1-2 times	< 1 time	Never	DK
Only sell at the market																		
Selling at other places																		
Using online/ phone (then deliver to consumers)																		
Others (specify): …………….																		

B7. At each below period, what is your <u>labor resource</u> and time involved in your ASF selling?

Content	At the same time last year *(before Feb/Mar 2020)*	During partial lockdown *(in weeks of April 2020)*	At present *(from May 2020 to now)*
**Labor** – number of people involve your ASF selling *(person)*	…..	…..	…..
**Time** – Total number of selling hours/day *(hour)*	…..	…..	…..

B8. Compared to before COVID-19 outbreak *(before Feb/Mar 2020, or at the same time last year)*, <u>how is your ASF selling income changed</u>? *(Filled the % accordingly if any)*

Compared period to	No change	Decreased (%)	Increased (%)	Don't know
**During partial lockdown** *(in weeks of April 2020)*	…..	…..	…..	
**At present** *(from May 2020 to now))*	…..	…..	…..	
	↓	↓	↓	↓
Move to	**C**	**B9**	**B10**	**C**

B9. What has caused *<u>decreased income</u>* from your selling? *(choose suitable -can choose more than 1 answers-)*

1. Markets closed/Seller were not allowed to sell due to partial lockdown
2. Cannot get ASF products to sell due to lack of ASF provider
3. Buyers go to market less frequency compared to before
4. Buyers bought less than before (e.g. due to higher price)
5. Other cheaper food types available
6. Other (specify): ……………
7. Do not know

B10. What has caused *<u>increased income</u>* from your selling? *(choose suitable answers)*

1. Increase number of customers, due to the ASF shops close
2. Customers buy more ASF in order to store the food
3. The customer buys more ASF (e.g. because the price is cheaper than other meat)
4. Due to increasing selling time per day (e.g. whole day instead of half day)
5. Other (specific): …………………………………
6. Do not know

C. KAP on COVID-19 and Prevention Measures

C1. Have you heard about COVID-19 and its pathogen?

1. Yes, due to virus
2. Yes, due to bacteria
3. Yes, due to parasite/protozoan
4. Yes, but don't know which pathogen
5. Neither heard about the disease nor know its pathogen

C2. How do you think COVID 19 spreads?

Content	Very likely	Possible, but not so likely	Not possible	Don't know
1. Through air				
2. Animal contact				
3. Through vector borne (insects: mosquitos, flies, ...)				
4. From a person who is sneezing and coughing				
5. From touching surfaces/objects with respiratory droplets from infected people				
6. From eating livestock products				
7. From eating wildlife products				
8. From contacting healthy people				

C3. What do you think is important to *stop the spread of* the disease?

Content	Very important	Little important	Not important	Don't know
1. Only staying at home				
2. Not meeting with strangers				
3. Avoid eating wildlife				
4. Avoid eating any animal sourced food				
5. Avoid eating any raw products (salad, fruit)				
6. Washing hands frequently				
7. Covering your mouth and nose while sneezing and coughing				
8. Avoid contact with people if you are having any symptoms				
9. Wear a mask when going out of the house				
10. Do social distancing when going out of the house				
11. Keeping people in quarantine after they returned from risky areas				
12. Keeping those people in quarantine who were in contact with an infected person				

C4. What are you doing to stop the disease yourself?

Content	I try to do this as much as possible	I do this sometimes	I do not do this	Not applicable
1. Only staying at home				
2. Not meeting with strangers				
3. Avoid eating wildlife				
4. Avoid eating any animal sourced food				
5. Avoid eating any raw products (salad, fruit)				
6. Washing hands frequently				
7. Covering your mouth and nose while sneezing and coughing				
8. Avoid contact with people if you are having any symptoms				
9. Wear a mask when going out of the house				
10. Do social distancing when going out of the house				

D. Food Safety Practices and Behaviors

D1. Frequency of food safety practices

Food safety practices	At the same time last year *(before Feb/Mar 2020)*					During partial lockdown *(in weeks of April 2020)*					At present *(from May 2020 to now)*				
	Very often	Often	Some times	Rarely	Never	Very often	Often	Some times	Rarely	Never	Very often	Often	Some times	Rarely	Never
1. Frequency of washing/cleaning your stall/shop															
2. Frequency of disinfection your stall/shop															
3. Frequency of washing your hands															
4. Frequency of disinfection your hands															
5. Wearing gloves?															
6. Wearing mask?															
7. Your ASF is checked for quality by authorities															
8. Eating/drinking at shop															
9. Keep fresh ASF at cool facility or on ice															

D2. Food safety perception and behavior

Items on food safety perception	Strongly agree	Agree	Neutral	Disagree	Strongly disagree	Don't know
1. Washing/cleaning shop equipment, facilities reduce my health risk						
2. Disinfection shop equipment and facilities is necessary						
3. Washing and disinfection my hands can reduce my health risk						
4. Washing and disinfection my hands during selling ASF is necessary						
5. Wearing mask can reduce my health risk						
6. Wearing mask is necessary						
7. Food inspection for quality and safety by authorities is necessary						
8. Seller health check is important						
9. My consumers are concerned my health (and not happy) when I wear mask during selling?						
10. My consumers concerning my health (and not happy) when I wear gloves during selling?						

Thank for your participation!
