# Supplementary material for: Impacts of the Pandemic, Animal Source Food Retailers’ and Consumers’ Knowledge and Attitudes toward COVID-19, and Their Food Safety Practices in Chiang Mai, Thailand
Source: Int J Environ Res Public Health. 2022 Aug 17;19(16):10187. doi: 10.3390/ijerph191610187 (PMC9408671; doi:10.3390/ijerph191610187)
Supplement: Supplementary file 1 [file ijerph-19-10187-s001.zip › Supplementary Files/Supplementary File S2 QX_COVID-19-consumers.pdf]

Survey questionnaires [ID.02-Consumers]: Assessing impacts of COVID-19 pandemic on animal source food retailers and consumers in Thailand (English translation)

A. Participant Information

A1. Gender:
1. Male
2. Female

A2. Year of birth: …………… (e.g. 1980)

A3. Sub-district: …………………….……… **District:** ……………………….

A4. Area:
1. Urban/Peri-urban
2. Rural

A5. What is your highest education level:
1. Illiterate
2. Elementary school
3. Secondary school
4. High school
5. Colleges, university, or higher degrees

A6. Occupation:
1. Farmers
2. Government officer/staff
3. Worker
4. Private business
5. Housewife
6. Students
7. Other……………

A7. What is your monthly family income in the last calendar year? By average family income -- income from all members in the family from all sources such as wages, self-employment and farming.

1. ≤ 6,000 THB	≤ 200 USD
2. 6,000 - 12,000 THB	200 - 400 USD
3. 12,000 - 18,000 THB	400 - 600 USD
4. 18,000 - 24,000 THB	600 - 800 USD
5. 24,000 - 30,000 THB	800 - 1,000 USD
6. 30,000 - 60,000 THB	1,000 - 2,000 USD
7. > 60,000 THB	> 2,000 USD
8. Not sure	Not sure

A8. Number of members who permanently stay in your family: ……… *(person)*

B. Impact of COVID-19 to ASF Purchasing and Selection

B1. At each below period, how often do you purchase ASF types for your home consumption? (Unit: times per week) *<ask all ASF if they consume>*

**ASF types** *(any applicable)*	**At the same time last year** *(before Feb/Mar 2020)*						**During partial lockdown** *(in weeks of April 2020)*						**At present** *(from May 2020 to now)*					
	Daily	4-5 times	2-3 times	One or < 1 times	Never	Don't know	Daily	4-5 times	2-3 times	One or < 1 times	Never	Don't know	Daily	4-5 times	2-3 times	One or < 1 times	Never	Don't know
Pork																		
Poultry (chicken, duck, ...)																		
Beef																		
Fish/seafood																		
Egg																		
Any kind of wildlife																		

B2. At each below period, which type of retail(s) have you often purchased ASF for your home consumption? *(time/week)*

**ASF types** *(any applicable)*	**At the same time last year** *(before Feb/Mar 2020)*						**During partial lockdown** *(in weeks of April 2020)*						**At present** *(from May 2020 to now)*					
	Daily	4-5 times	2-3 times	One or < 1 times	Never	Don't know	Daily	4-5 times	2-3 times	One or < 1 times	Never	Don't know	Daily	4-5 times	2-3 times	One or < 1 times	Never	Don't know
Traditional market																		
Wet market																		
Super market																		
Convenience store																		
Street vendor																		
Your own																		
Online (ship)																		

B3. At each below period, what is an average amount of ASF type (Kg) have you purchase per shopping time? (If they don't eat => **NA**; don't know/don't remember, put "**DK**")

**ASF types** *(any applicable)*	**At the same time last year** *(before Feb/Mar 2020)*	**During partial lockdown** *(in weeks of April 2020)*	**At present** *(from May 2020 to now)*
Pork	.....	.....	.....
Poultry (chicken, duck, ...)	.....	.....	.....
Beef	.....	.....	.....
Fish/seafood	.....	.....	.....
Egg*	.....	.....	.....
Any kind of wildlife	.....	.....	.....

**Number of eggs/time (chicken, duck eggs)*

B4. At present *(from May 2020 to now)*, <u>How easy</u> for you to buy ASF at the time of <u>COMPARED</u> to before COVID-19 or at the same time last year? *(before Feb/Mar 2020)*

ASF types	Easier	No change/normal	More difficult	Don't know
Pork				
Poultry (chicken, duck, ...)				
Beef				
Fish/seafood				
Egg				
Any kind of wildlife				

B5. During partial lockdown *(in weeks of April 2020)*, <u>How easy</u> for you to buy ASF at the time of <u>COMPARED</u> to before COVID-19 or at the same time last year? *(before Feb/Mar 2020)*

ASF types	Easier	No change/normal	More difficult	Don't know
Pork				
Poultry (chicken, duck, ...)				
Beef				
Fish/seafood				
Egg*				
Any kind of wildlife				

* Number of eggs/time (chicken, duck egg, ...)

C. Impact of COVID-19 to Consumer's Job and Livelihood

C1. Did COVID-19 impact to your business/job/work/employment?

1. Yes, increase (→ **C2, C4, C5**)
2. Yes, decrease (→ **C2, C3, C5**)
3. No (**Move to** → **C5**)
4. Don't know (**Move to** → **C5**)

C2. If Yes, how much has your income/salary *<u>increased/decreased</u>*?

1. All or nearly all (90-100%)
2. More than half of it (60-90%)
3. About half of it (40-60%)
4. Less than half of it (10-40%)
5. A small amount (1-10%)
6. Don't know / no answer

C3. What has caused *<u>decreased</u>* income your business/job?

1. Reduced mobility / enforced lockdown
2. Increased duty of care on children or sick
3. Reduced work time
4. Loss job
5. Other (specify): ………………
6. Don't know / no answer

C4. What has caused *<u>increased</u>* income your business/job? ………………………………

C5. Think back before, during the COVID-19 periods and this time

Items	At the same time last year *(before Feb/Mar 2020)*			During partial lockdown *(in weeks of April 2020)*			At present *(from May 2020 to now)*		
	Yes	No	Don't know	Yes	No	Don't know	Yes	No	Don't know
1. You or others in the household were worried about not having enough food to eat because of a lack of money or other resources?									
2. You or others in your household were unable to eat healthy and nutritious food because of a lack of money or other resources?									
3. You or others in your household ate only a few kinds of foods because of a lack of money or other resources to get food?									
4. You or others in your household had to skip a meal because there was not enough money or other resources to get food?									
5. You or others in your household ate less than you thought you should because of a lack of money or other resources?									
6. Your household ran out of food because of a lack of money or other resources?									
7. You or others in your household were hungry but did not eat because there was not enough money or other resources for food?									
8. You or others in your household went without eating for a whole day because of a lack of money or other resources?									

D. KAP on COVID-19 and Prevention Measures

D1. Have you heard about COVID-19 and its pathogen?

1. Yes, due to virus
2. Yes, due to bacteria
3. Yes, due to parasite/protozoan
4. Yes, but don't know which pathogen
5. Neither heard about the disease nor know its pathogen

D2. How do you think COVID-19 spreads?

Content	Very likely	Possible, but not so likely	Not possible	Don't know
1. Through air				
2. Animal contact				
3. Through vector borne (insects: mosquitos, flies...)				
4. From a person who is sneezing and coughing				
5. From touching surfaces/objects with respiratory droplets from infected people				
6. From eating livestock products				
7. From eating wildlife products				
8. From contacting healthy people				

D3. What do you think is important to *stop the spread of* the disease?

Content	Very important	Little important	Not important	Don't know
1. Only staying at home				
2. Not meeting with strangers				
3. Avoid eating wildlife				
4. Avoid eating any animal sourced food				
5. Avoid eating any raw products (salad, fruit)				
6. Washing hands frequently				
7. Covering your mouth and nose while sneezing and coughing				
8. Avoid contact with people if you are having any symptoms				
9. Wear a mask when going out of the house				
10. Do social distancing when going out of the house				
11. Keeping people in quarantine after they returned from risky areas				
12. Keeping those people in quarantine who were in contact with an infected person				

D4. What are you doing to stop the disease yourself?

**Content**	I try to do this as much as possible	I do this sometimes	I do not do this	Not applicable
1. Only staying at home				
2. Not meeting with strangers				
3. Avoid eating wildlife				
4. Avoid eating any animal sourced food				
5. Avoid eating any raw products (salad, fruit)				
6. Washing hands frequentlyz				
7. Covering your mouth and nose while sneezing and coughing				
8. Avoid contact with people if you are having any symptoms				
9. Wear a mask when going out of the house				
10. Do social distancing when going out of the house				

E. Food Safety Practices and Behaviors

E1. Before or during COVID-19 periods, how often do you do practices as below?

Practices *(any applicable)*	At the same time last year *(before Feb/Mar 2020)*					During partial lockdown *(in weeks of April 2020)*					At present *(from May 2020 to now)*				
	Very often	Often	Some times	Rarely	Never	Very often	Often	Some times	Rarely	Never	Very often	Often	Some times	Rarely	Never
1. Seeking ASF with proved quality check and clear origin															
2. Wash your ASF carefully before cooking															
3. Frequency of eaten rare or undercook ASF															
4. Frequency of washing kitchen equipment after preparing ASF															
5. Frequency of washing your hands after preparing ASF															
6. Using separate kitchen utensils for raw and cooked ASF															
7. Wearing gloves when you prepare raw ASF															
8. Disinfection hands after returning home from going out															

E2. Food safety perception and behavior - What is your opinion about below statements?

Items on food safety perception	Strongly agree	Agree	Neutral	Disagree	Strongly disagree	Don't know
1. I am looking for a good hygiene practice shop to buy ASF						
2. I expect sellers to use a mask when selling ASF						
3. I expect sellers to use a glove when selling ASF						
4. Washing/cleaning kitchen equipment, facilities frequency reduces my health risk						
5. Washing and disinfection frequently hands reduce my health risk						
6. Food inspection for quality and safety by authorities is necessary						
7. Shop sellers wear mask during selling is to hide their health problem						
8. Shop sellers wear gloves during selling is to hide their health problem						
9. I avoid eating raw or undercooked ASF						

Thank for your participation!
