# Supplementary material for: Impacts of the Pandemic, Animal Source Food Retailers’ and Consumers’ Knowledge and Attitudes toward COVID-19, and Their Food Safety Practices in Chiang Mai, Thailand
Source: Int J Environ Res Public Health. 2022 Aug 17;19(16):10187. doi: 10.3390/ijerph191610187 (PMC9408671; doi:10.3390/ijerph191610187)
Supplement: Supplementary file 1 [file ijerph-19-10187-s001.zip › Supplementary Files/Supplementary File S3 Figures and tables.pdf]

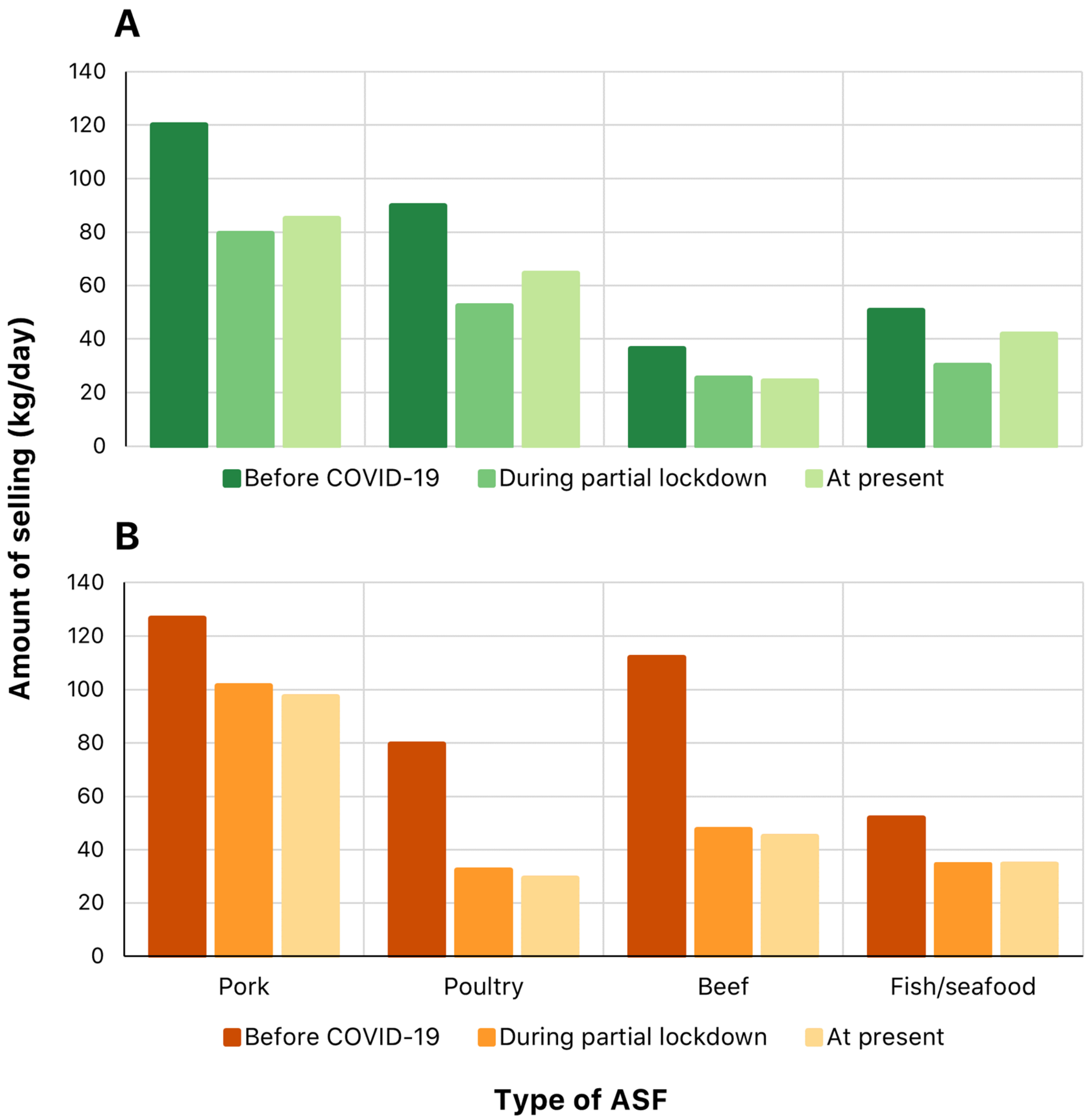

Figure S1. Average amounts of ASF sold at different periods in (**A**) U/PU and (**B**) rural areas.

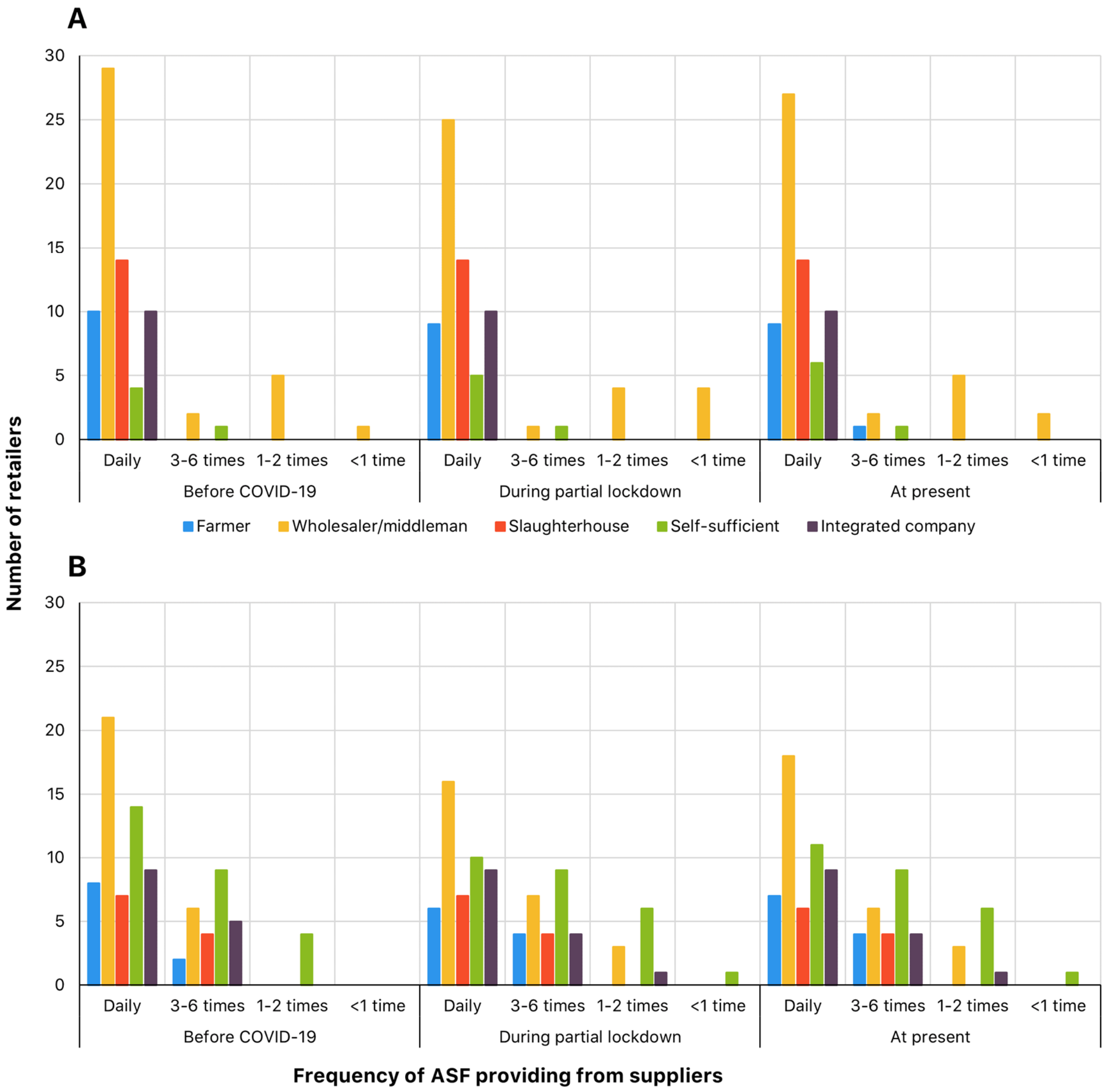

Figure S2. Frequency of ASF provided by suppliers in (**A**) U/PU and (**B**) rural areas.

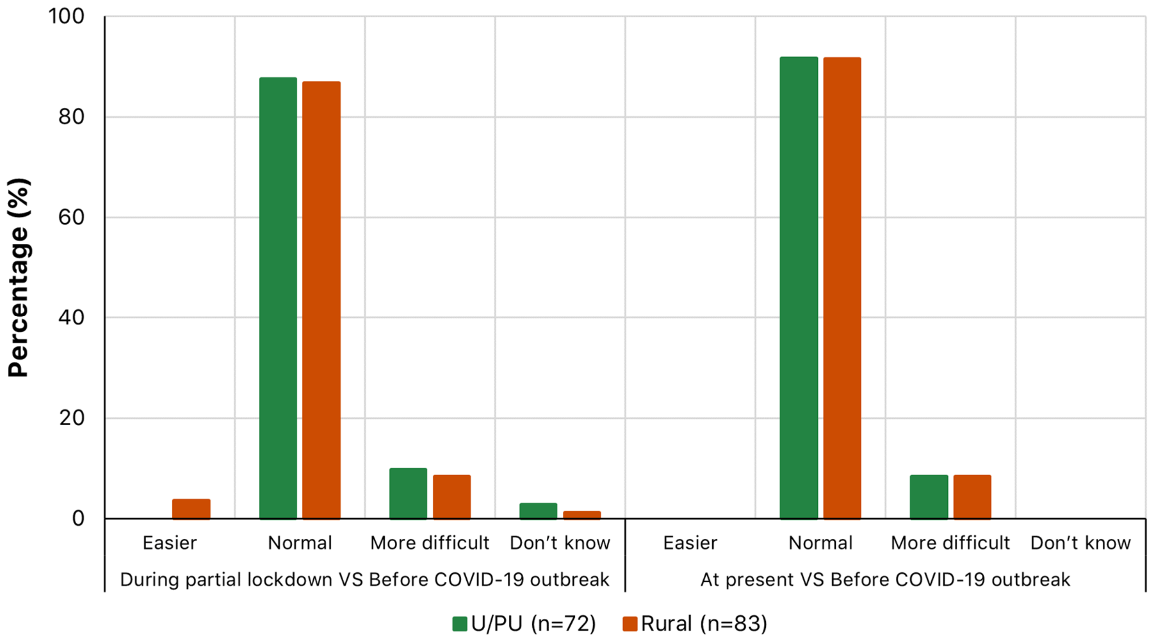

Figure S3. Difficulties of providing ASF to sell in different periods compared with the time before an outbreak.

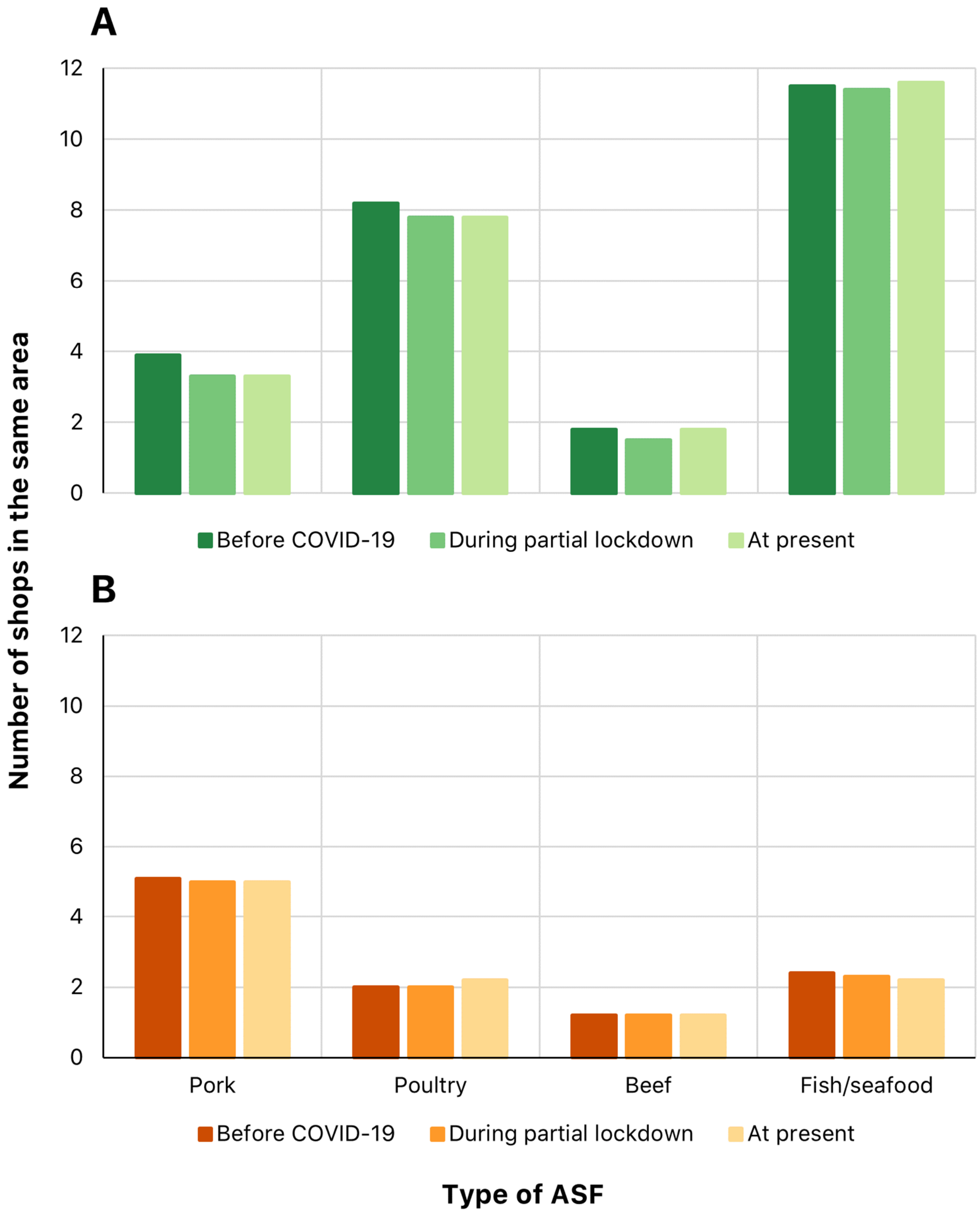

Figure S4. Average numbers of ASF shops in the same market in (**A**) U/PU and (**B**) rural areas.

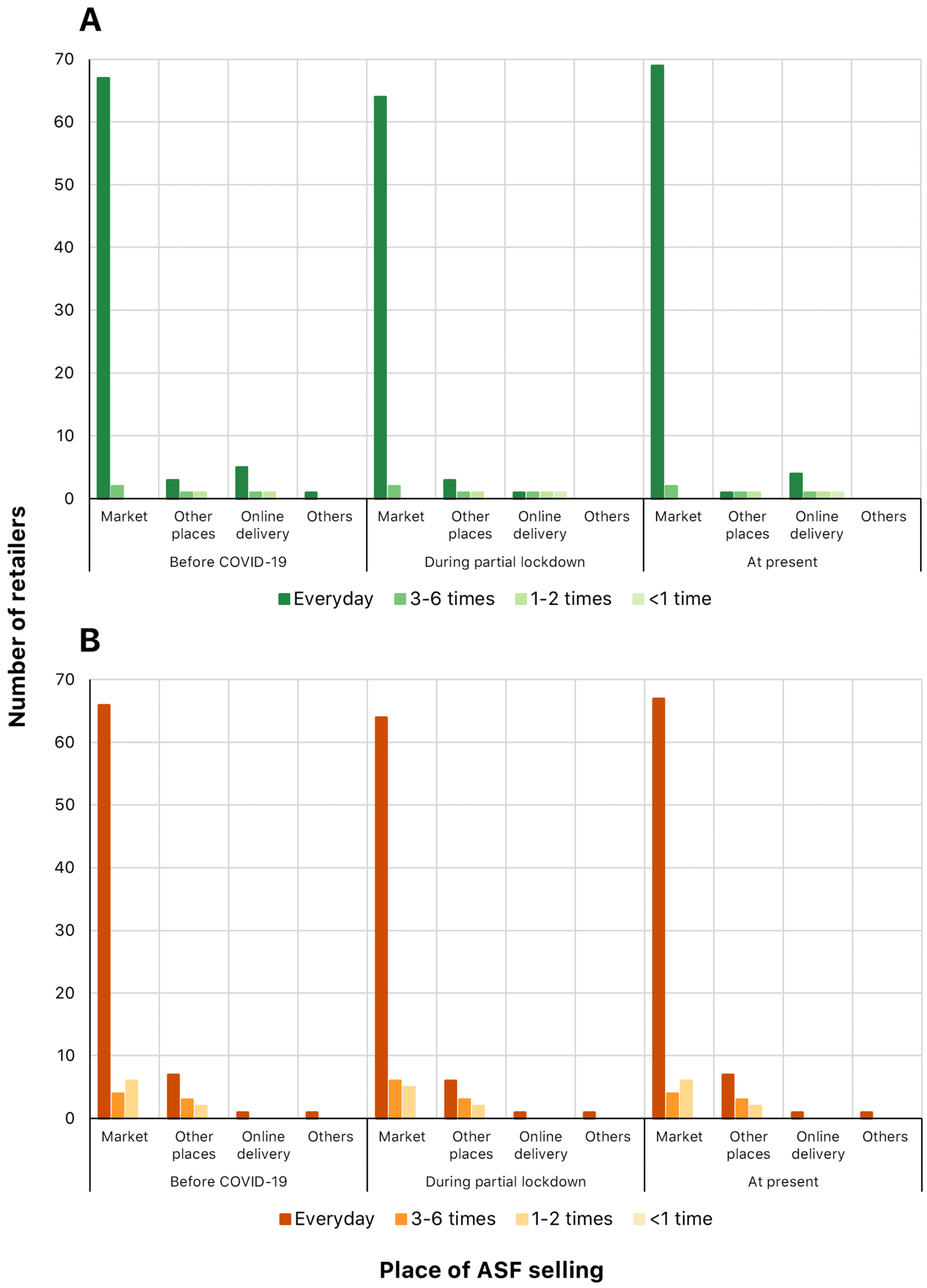

Figure S5. Locations and frequency of ASF selling by retailers in (**A**) U/PU and (**B**) rural areas. Other places included flea market, their own shops, food stall beside the road, etc. Others were listed as alternative way of selling, such as distributing to restaurants.

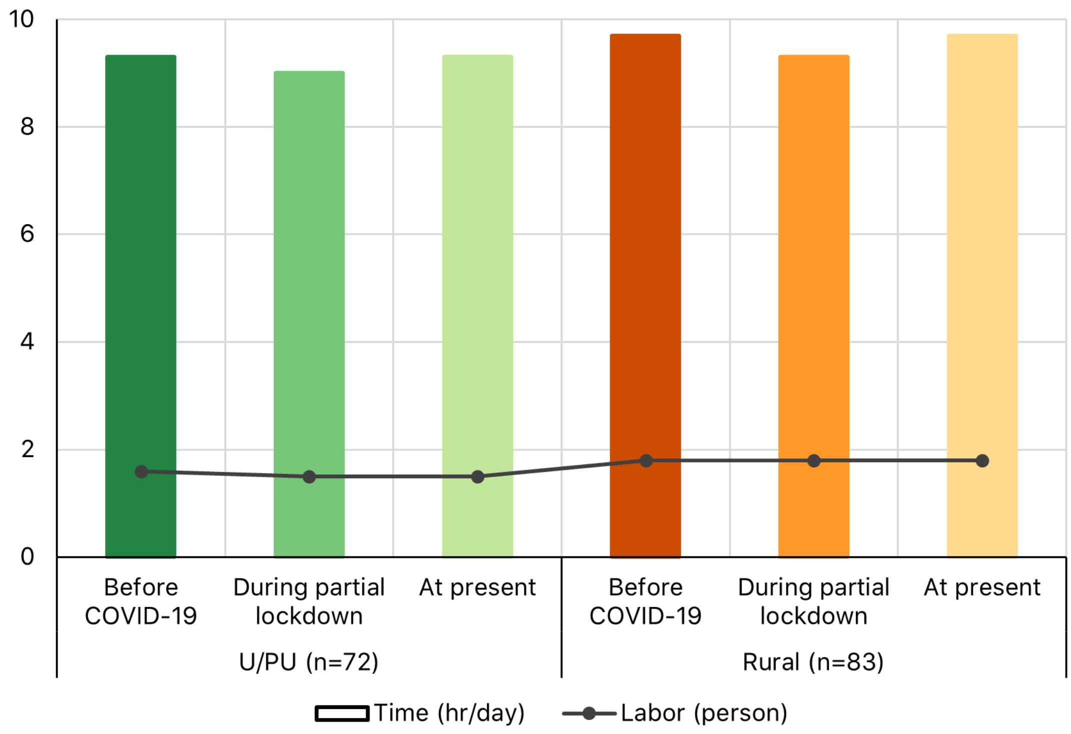

Figure S6. Labor resource and time spent on ASF selling.

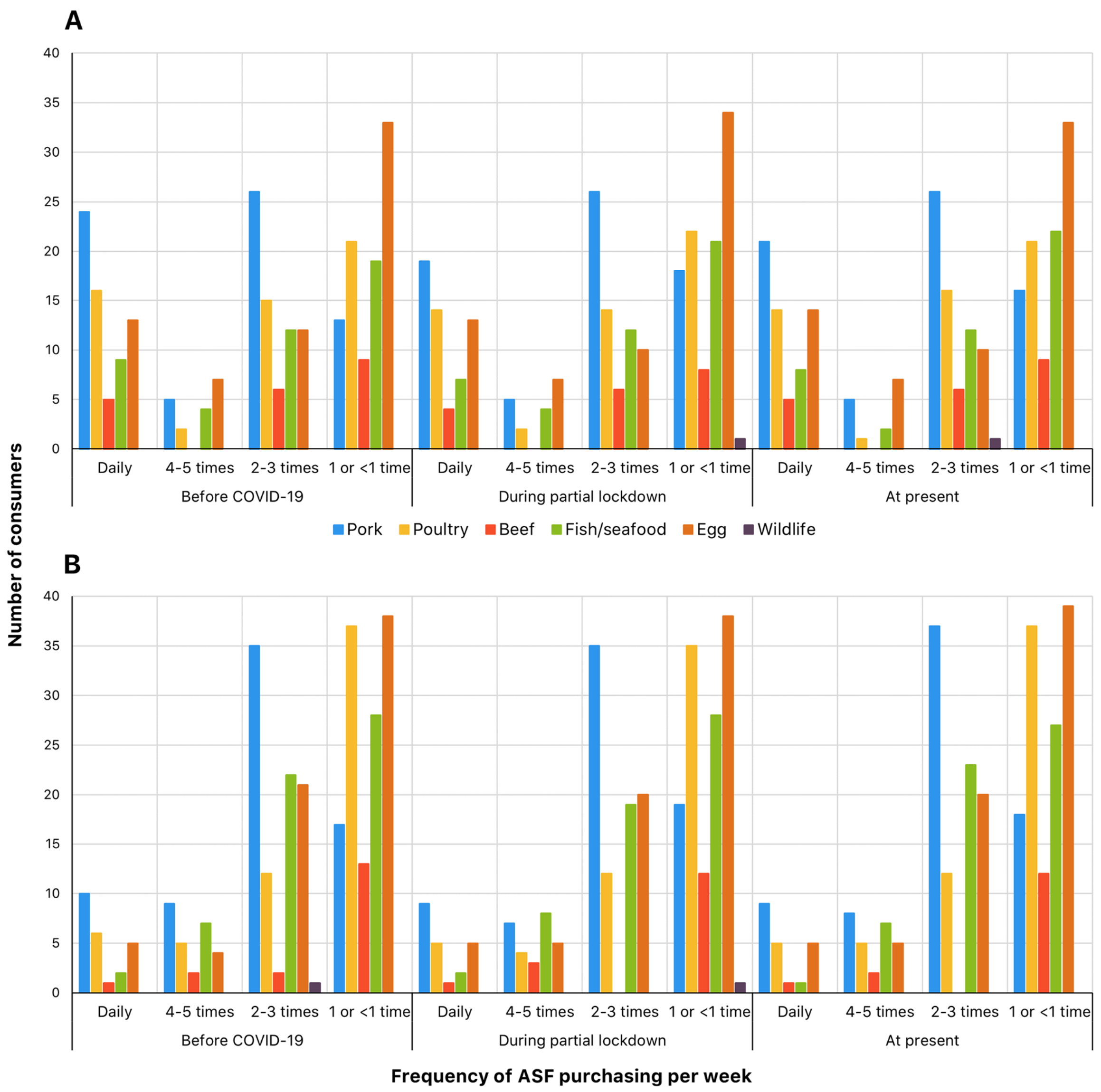

Figure S7. Impacts of COVID-19 to ASF purchasing and selection of the consumers in (**A**) U/PU and (**B**) rural areas.

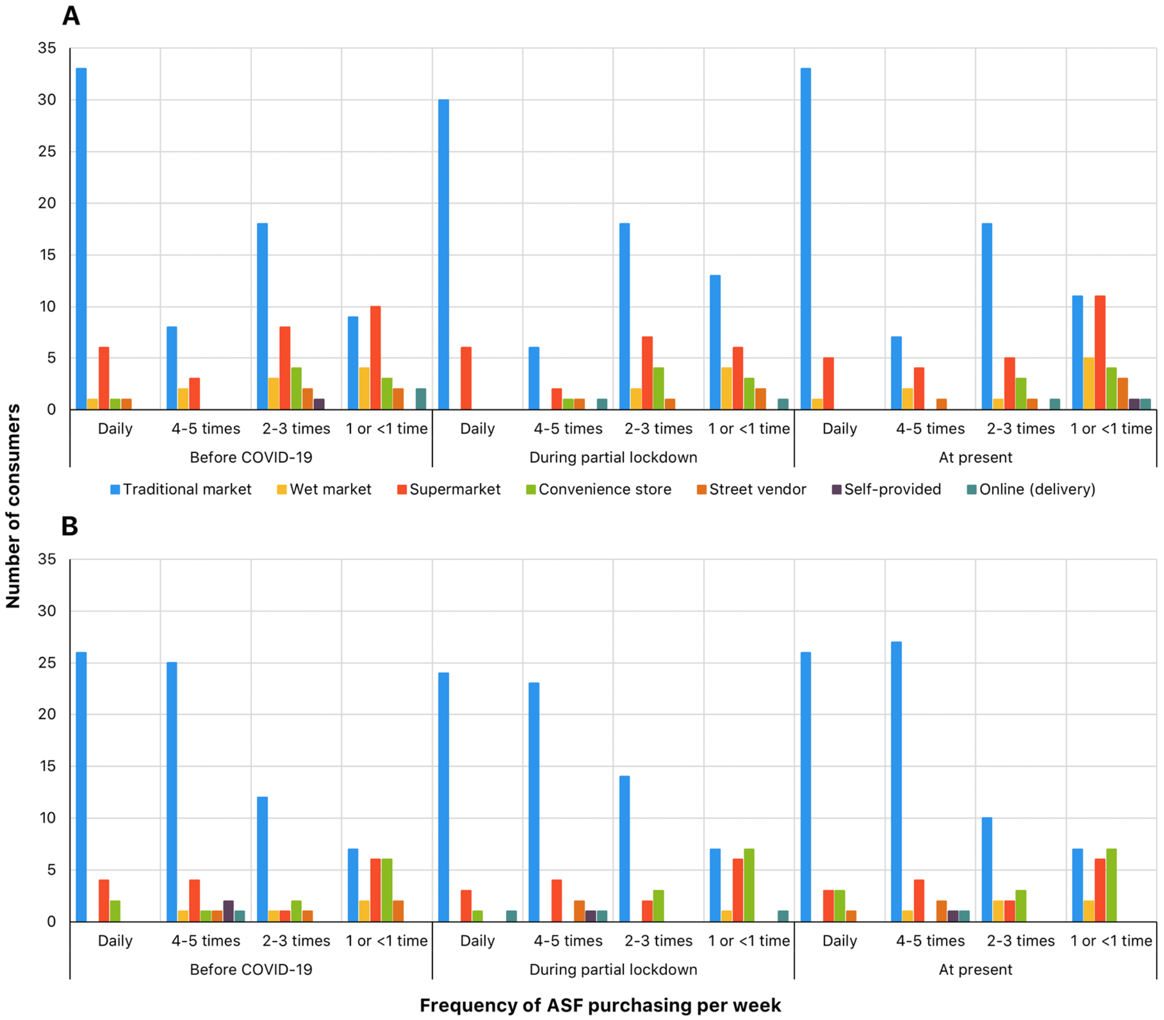

Figure S8. Preference type of retails and purchasing frequency of consumers in (**A**) U/PU and (**B**) rural areas. Self-provided, the consumers prepared ASF themselves (had their own farms or raised livestock in their households for consumption).

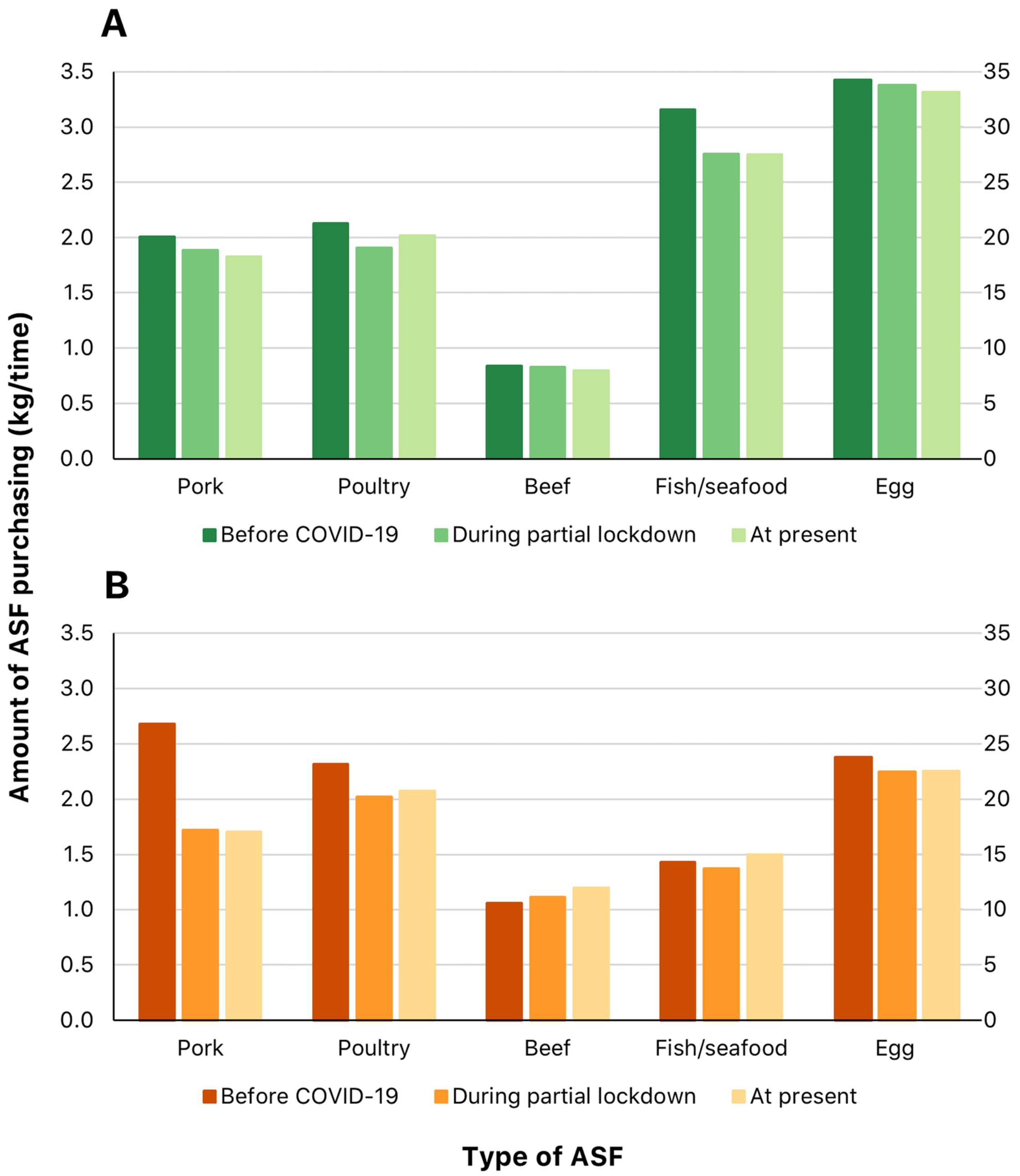

Figure S9. Average amount of ASF purchased (kg) per shopping trip of consumers in (**A**) U/PU and (**B**) rural areas. Amount of eggs purchasing was referred to the secondary (right) axis (eggs/time).

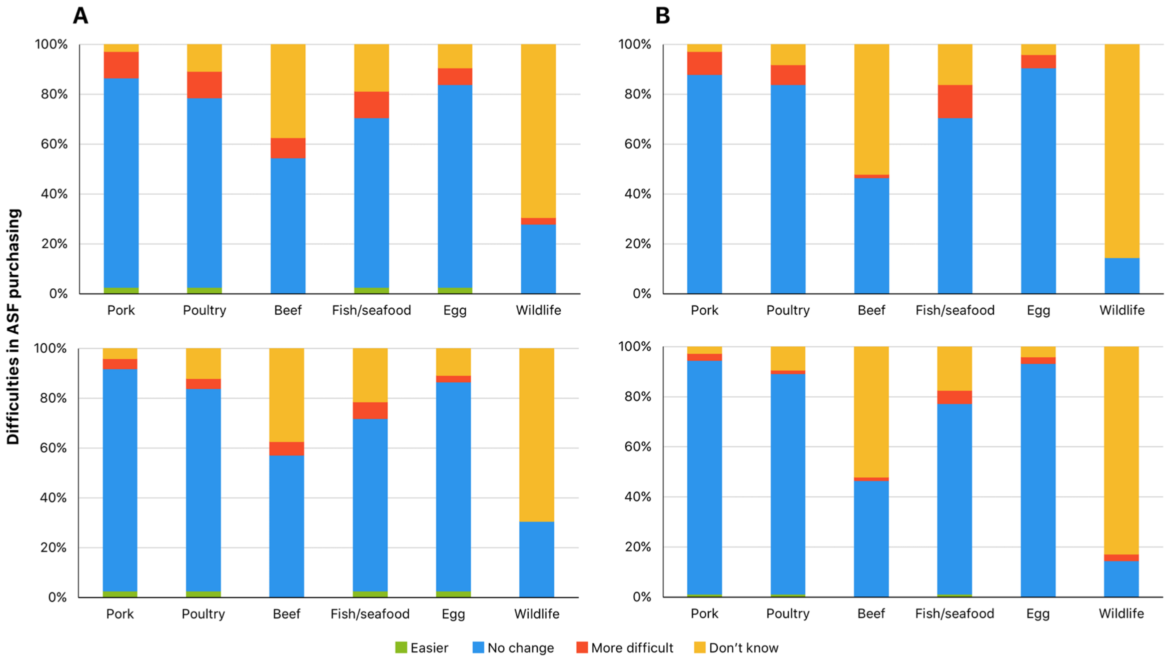

Figure S10. Difficulties of consumers in ASF purchasing; (**A**) U/PU areas (**A-Upper**) during partial lockdown and (**A-Lower**) at present, compared with the time before COVID-19 outbreak; (**B**) rural areas (**B-Upper**) during partial lockdown and (**B-Lower**) at present, compared with the time before COVID-19 outbreak.

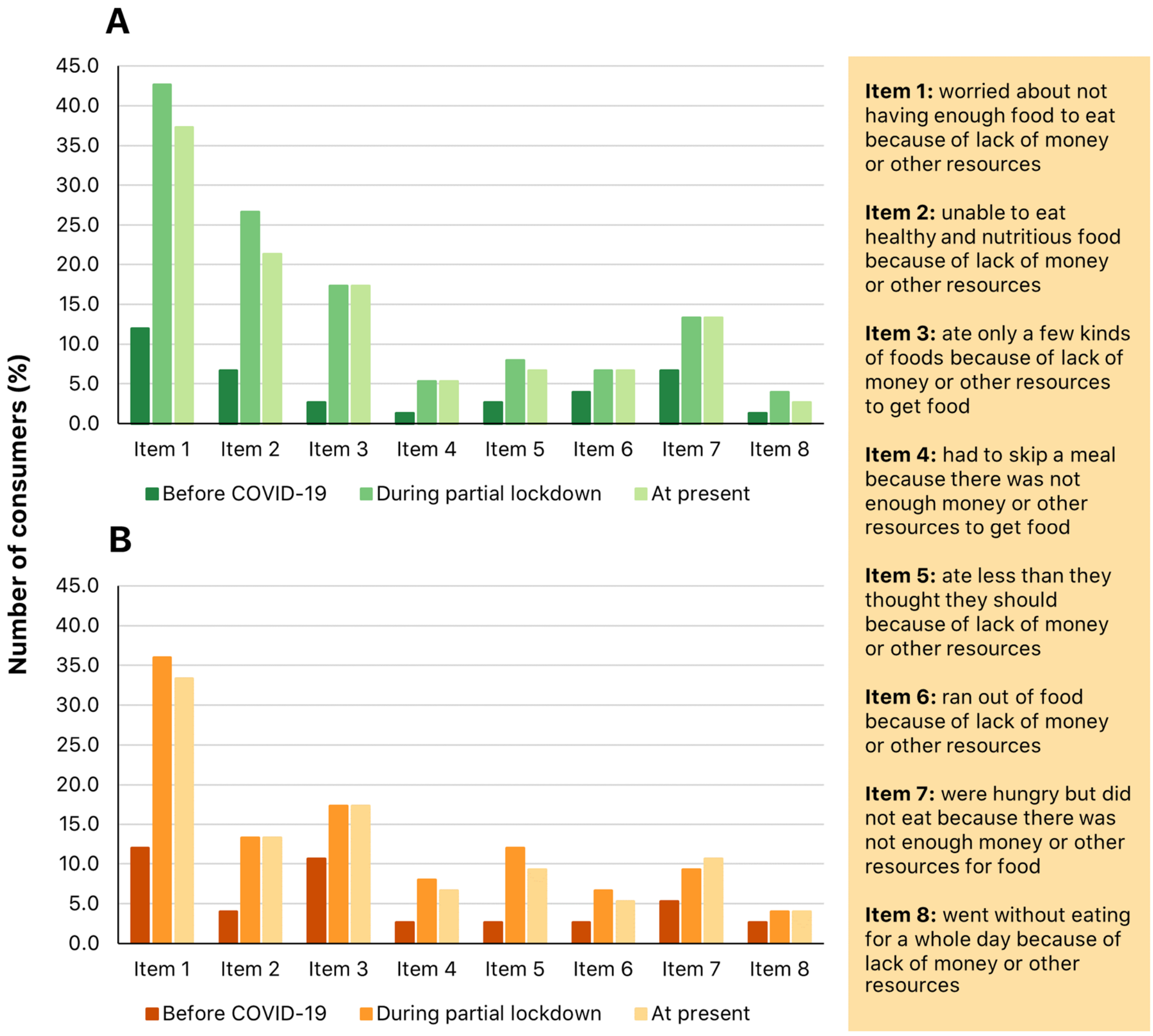

Figure S11. Impacts of COVID-19 on consumer livelihood in (**A**) U/PU and (**B**) rural areas.

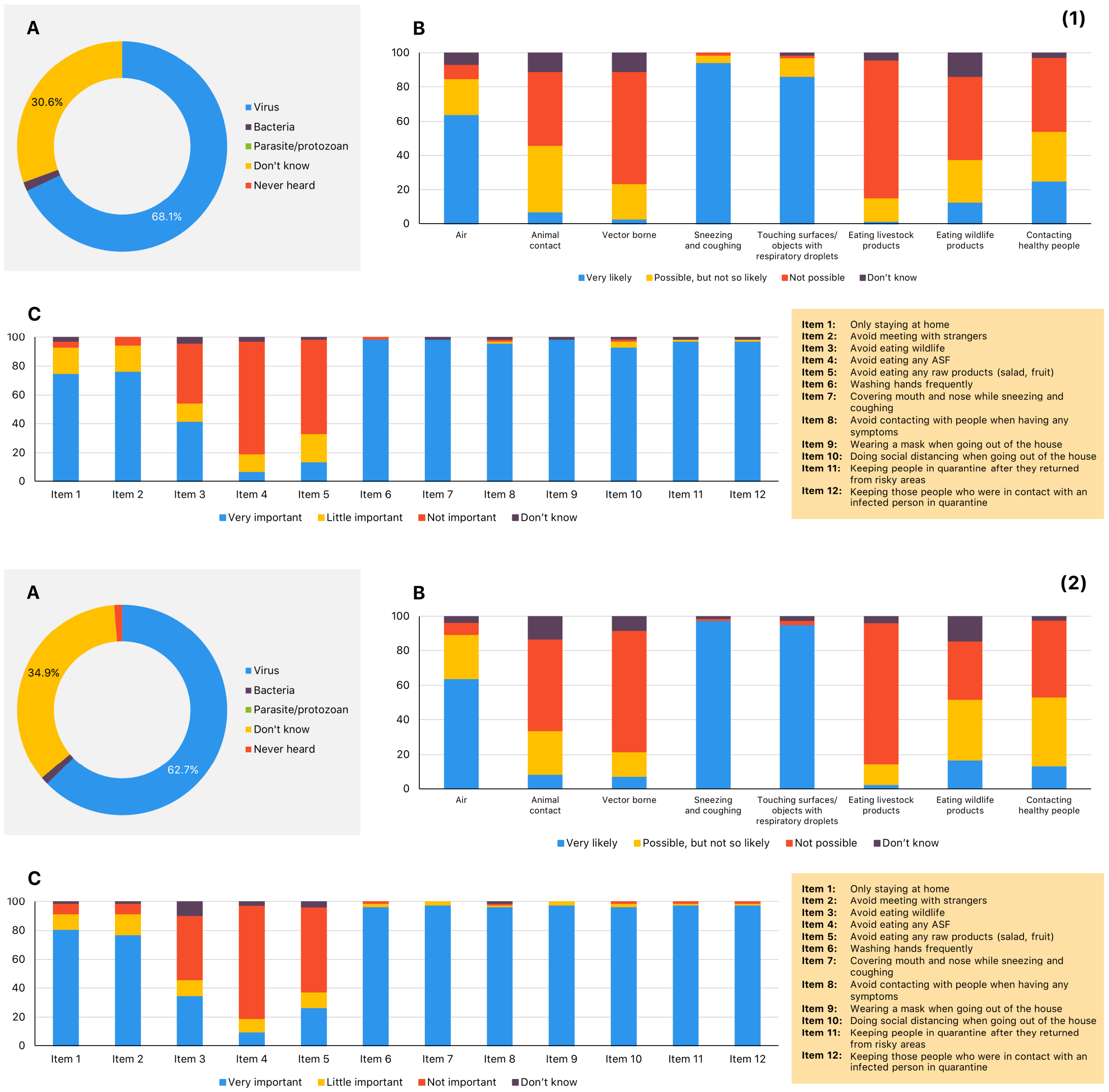

Figure S12. Knowledge of ASF retailers in (**1**) U/PU and (**2**) rural areas toward COVID-19 regarding (**A**) causative agent, (**B**) transmissions, and (**C**) preventive measures.

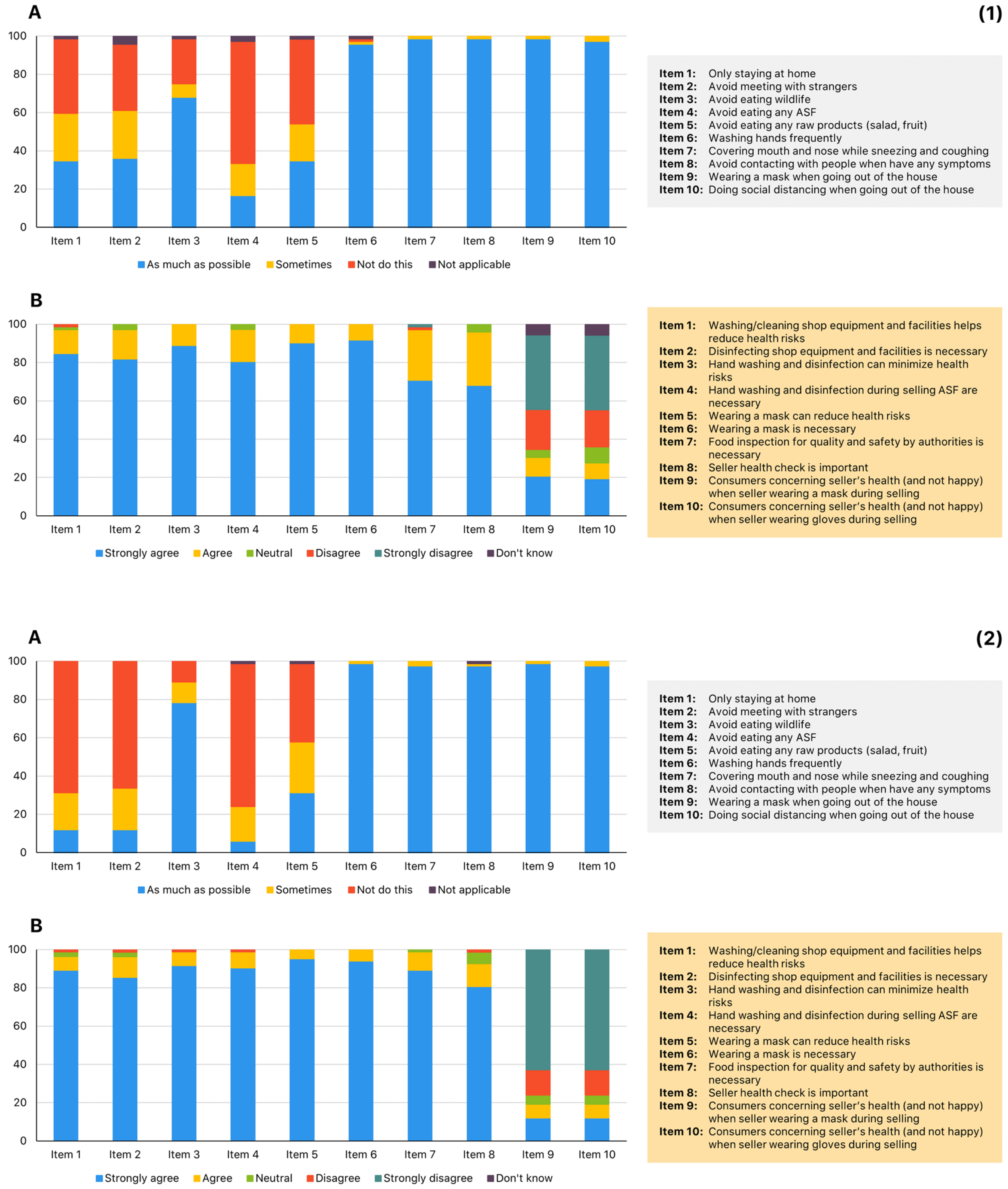

Figure S13. Attitudes of retailers in (**1**) U/PU and (**2**) rural areas regarding (**A**) COVID-19 prevention and (**B**) food safety practice.

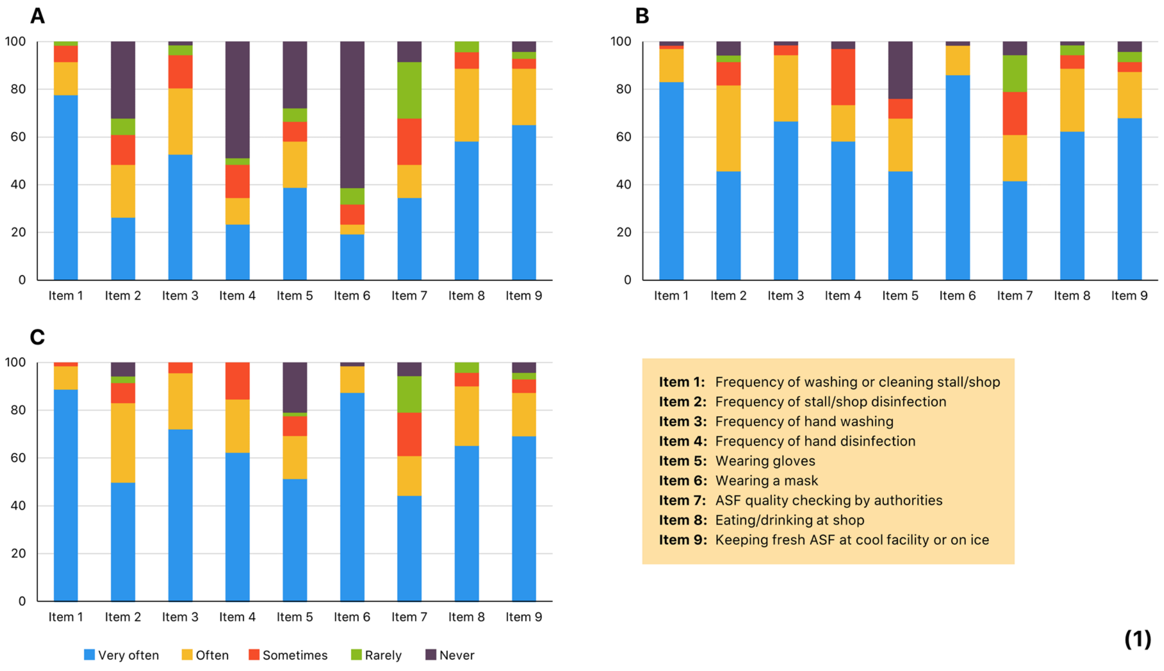

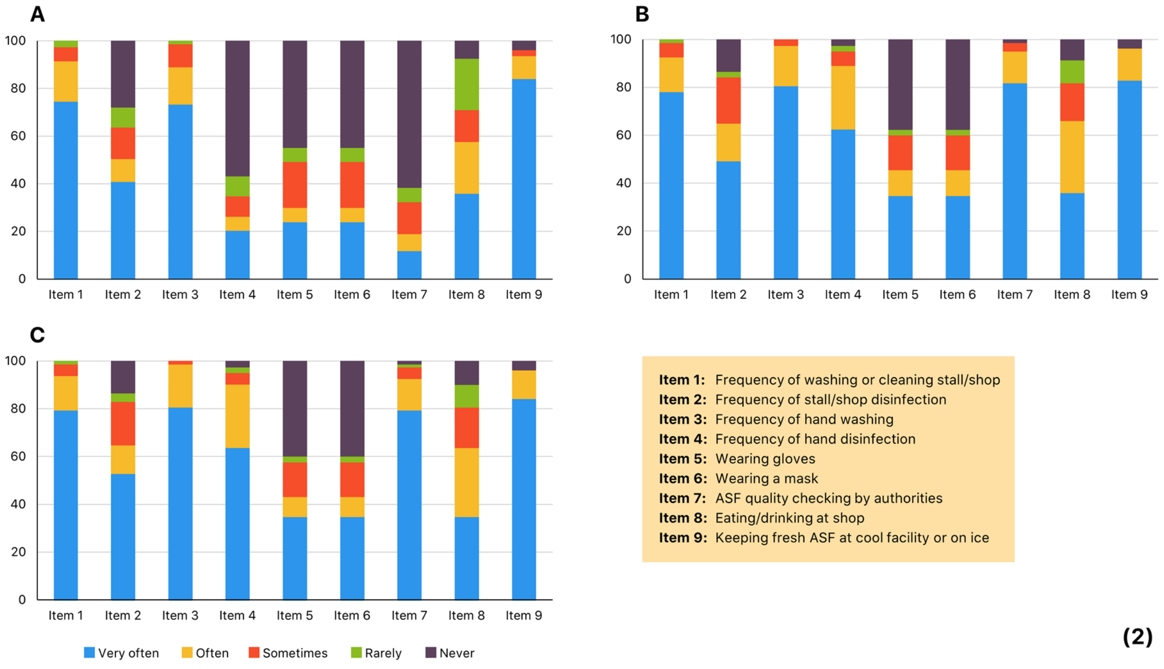

Figure S14. Practices toward food safety of retailers in (**1**) U/PU and (**2**) rural areas (**A**) at the time before COVID-19 outbreak, (**B**) during partial lockdown, and (**C**) at present.

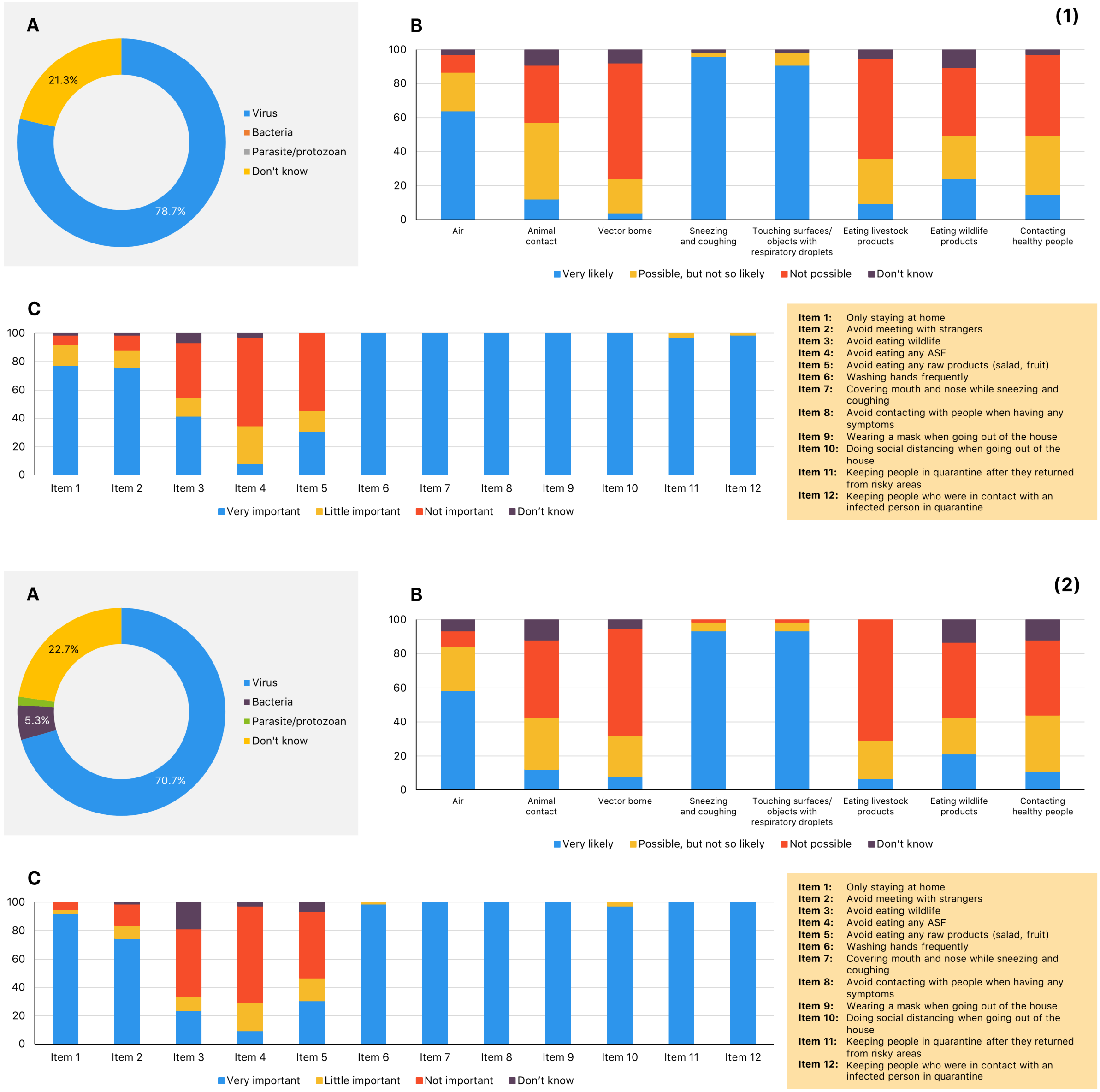

Figure S15. Knowledge of ASF consumers in (**1**) U/PU and (**2**) rural areas toward COVID-19 regarding (**A**) causative agent, (**B**) transmissions, and (**C**) preventive measures.

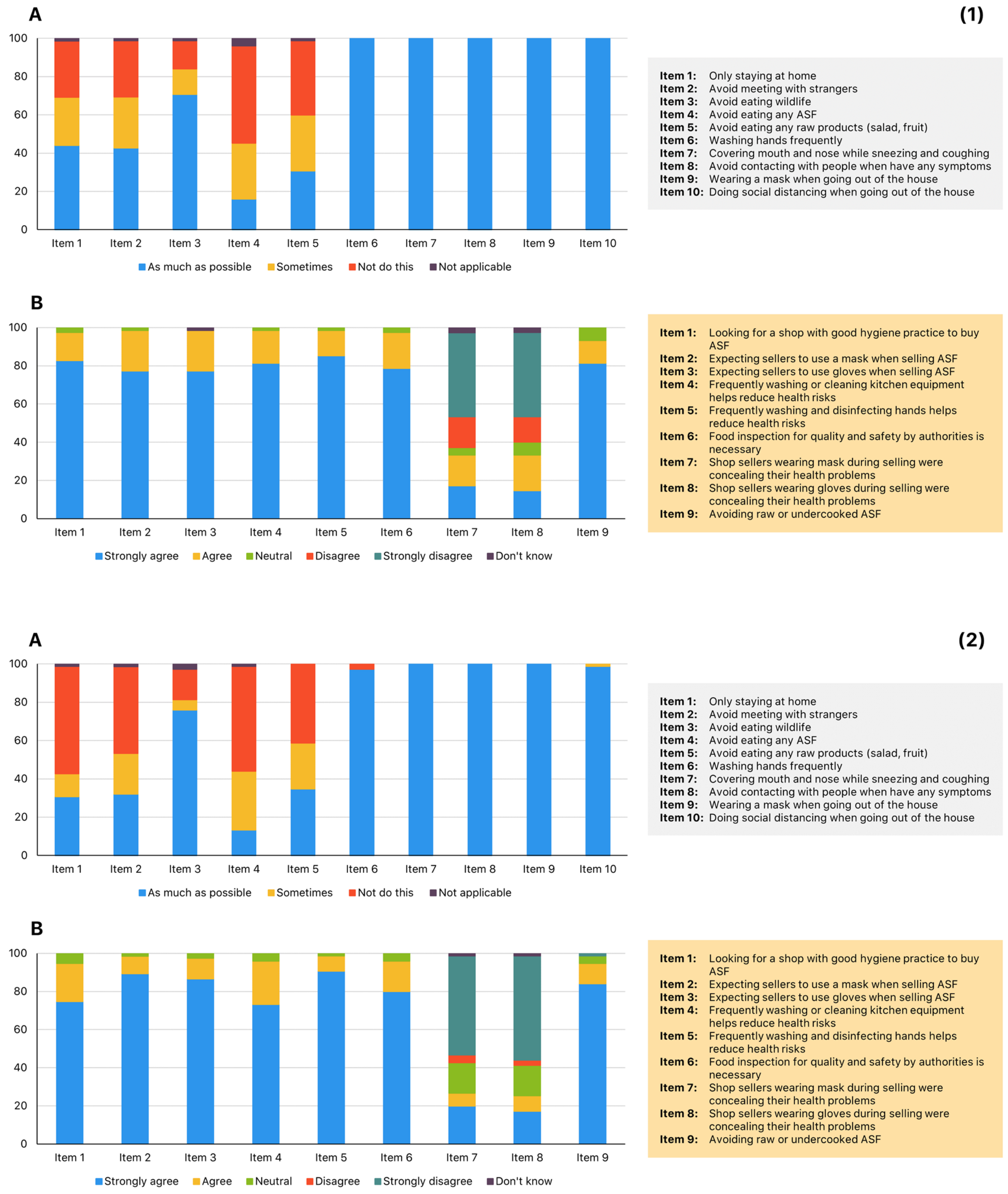

Figure S16. Attitudes of consumers in (**1**) U/PU and (**2**) rural areas regarding (**A**) COVID-19 prevention and (**B**) food safety practice.

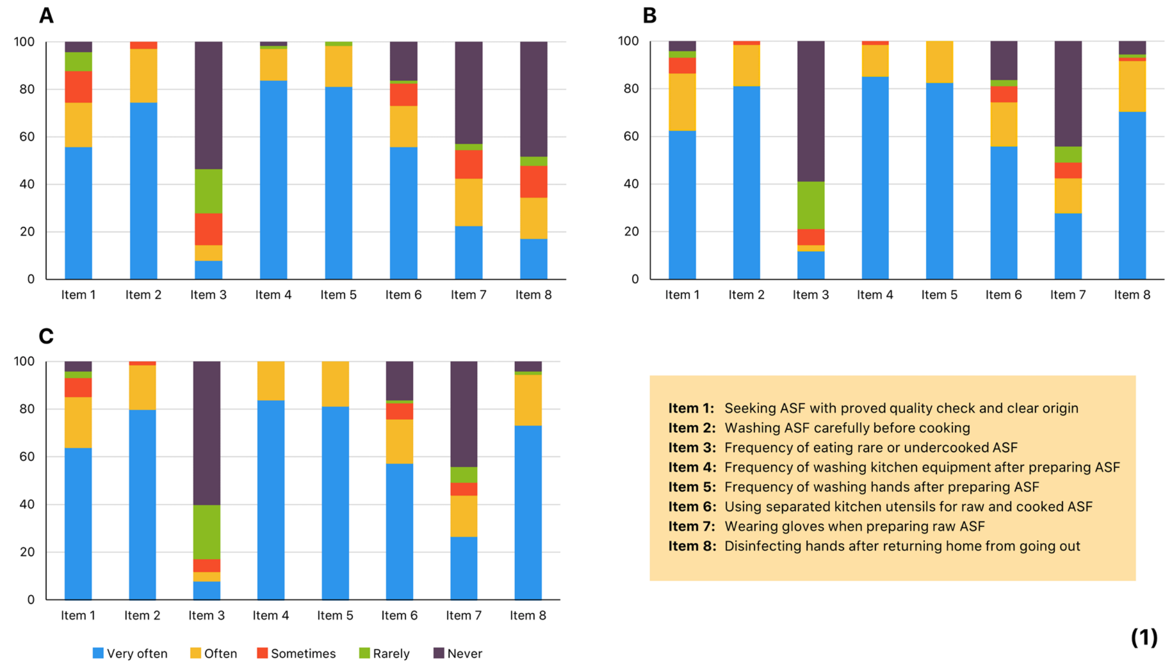

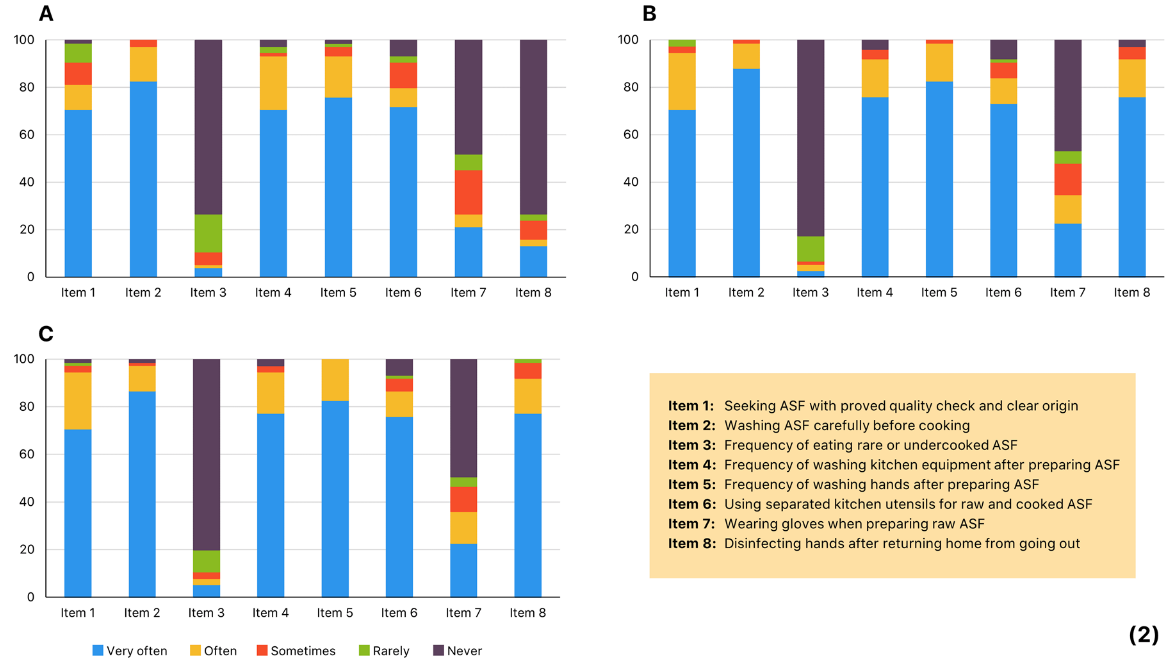

Figure S17. Practices toward food safety of consumers in (**1**) U/PU and (**2**) rural areas (**A**) at the time before COVID-19 outbreak, (**B**) during partial lockdown, and (**C**) at present.

Table S1. Differences of KAP scores between retailers in U/PU and rural areas.

Category	n	Mean difference ± SE	*p*-value	95% CI
**Knowledge toward COVID-19** [a]	155	0.06 ± 0.57	0.913	-1.07, 1.19
**Attitudes toward** [a]	155			
COVID-19 prevention		-1.75 ± 0.42 [b]	< 0.001	-2.58, -0.92
food safety practice		-2.06 ± 0.66	0.002	-3.36, - 0.76
**Practices toward food safety** [a]	155			
before COVID-19 outbreak		0.05 ± 0.96	0.960	-1.85, 1.95
during partial lockdown		0.29 ± 0.78	0.713	-1.25, 1.82
at present		0.98 ± 0.68	0.151	-0.36, 2.33
**Practices of retailers in U/PU**	72			
Before COVID-19 *VS* during lockdown		-6.61 ± 0.66 [c]	< 0.001	-7.93, -5.29
Before COVID-19 *VS* at present		-7.24 ± 0.59	< 0.001	-8.41, -6.06
During lockdown *VS* at present		-0.63 ± 0.35	0.078	-1.32, 0.07
**Practices of retailers in rural**	83			
Before COVID-19 *VS* during lockdown		-6.37 ± 0.46	< 0.001	-7.28, -5.46
Before COVID-19 *VS* at present		-6.30 ± 0.47	< 0.001	-7.23, -5.37
During lockdown *VS* at present		0.07 ± 0.16	0.661	-0.25, 0.40

[a] Mean difference of KAP scores were used the scores of U/PU retailers as minuends.

[b] Negative value of mean difference indicated the rural mean score was higher than the U/PU for that category or

[c] the latter category scored higher than the first category for comparison of food safety practices.

Table S2. Differences of KAP scores between consumers in U/PU and rural areas.

Category	n	Mean difference ± SE	*p*-value	95% CI
**Knowledge toward COVID-19** [a]	150	0.59 ± 0.41	0.153	-0.22, 1.39
**Attitudes toward** [a]	150			
COVID-19 prevention		-0.77 ± 0.42 [b]	0.065	-1.59, 0.05
food safety practice		-0.41 ± 0.74	0.575	-1.87, 1.04
**Practices toward food safety** [a]	150			
before COVID-19 outbreak		1.15 ± 0.61	0.064	-0.07, 2.36
during partial lockdown		0.13 ± 0.59	0.823	-1.04, 1.31
at present		-0.13 ± 0.61	0.826	-1.33, 1.07
**Practices of consumers in U/PU**	75			
Before COVID-19 *VS* during lockdown		-2.32 ± 0.32	< 0.001	-2.96, -1.68
Before COVID-19 *VS* at present		-2.29 ± 0.32	< 0.001	-2.93, -1.66
During lockdown *VS* at present		0.03 ± 0.14	0.849	-0.25, 0.30
**Practices of consumers in rural**	75			
Before COVID-19 *VS* during lockdown		-3.33 ± 0.25	< 0.001	-3.84, -2.83
Before COVID-19 *VS* at present		-3.57 ± 0.27	< 0.001	-4.11, -3.04
During lockdown *VS* at present		-0.24 ± 0.15	0.106	-0.53, 0.05

[a] Mean difference of KAP scores were used the scores of U/PU consumers as minuends.
[b] Negative value of mean difference indicated the rural mean score was higher than the U/PU for that category or
[c] the latter category scored higher than the first category for comparison of food safety practices.

Table S3. Multivariable analysis results of retailers.

Final generalized linear models (GLM) and variables [a]	$OR_{adjusted}$	95%CI	*p*-value
**GLM 1: Factors relevant to good knowledge toward COVID-19 (n = 154)**			
**Gender**			
Male	1		
Female	**2.99** [c]	**1.27, 7.02**	**0.012**
**Age group**			
20 – 29	1		
30 – 39	0.35	0.06, 2.05	0.246
40 – 49	0.51	0.09, 3.02	0.457
50 – 60	0.78	0.13, 4.86	0.791
> 60	0.68	0.10, 4.56	0.693
**Education**			
Illiterate	1		
Primary school	0.97	0.22, 4.19	0.968
Secondary school	3.13	0.37, 26.86	0.298
High school	2.29	0.42, 12.50	0.340
College or higher	3.68	0.66, 20.36	0.136
**Type of ASF**			
Pork	1		
Poultry	1.26	0.42, 3.73	0.678
Beef	3.05	0.52, 17.71	0.215
Fish/seafood	2.07	0.77, 5.60	0.150
**Business type** [b]			
Retail only	1		
Wholesale and retail	3.16	0.92, 10.85	0.067
**GLM 2: Factors relevant to good practices toward food safety (n = 155)**			
**Age group**			
20 – 29	1		
30 – 39	**5.07**	**1.34, 19.15**	**0.017**
40 – 49	1.78	0.52, 6.12	0.361
50 – 60	2.57	0.71, 9.34	0.151
> 60	1.02	0.25, 4.21	0.975
**Education**			
Illiterate	1		
Primary school	2.08	0.50, 8.63	0.314
Secondary school	1.12	0.17, 7.36	0.907
High school	2.53	0.53, 12.05	0.243
College or higher	1.07	0.24, 4.86	0.930
**Type of ASF**			
Pork	1		
Poultry	**0.36**	**0.14, 0.96**	**0.040**
Beef	0.33	0.09, 1.29	0.112
Fish/seafood	**0.22**	**0.09, 0.54**	**0.001**

OR, odds ratio; CI, confidence interval. Value of 1 was the category used as a reference for the comparable category.

[a] Only variables with *p*-value of less than 0.2 were included in the GLM. There was no factor significantly related to attitudes toward COVID-19 prevention. Business type of retailers was the only factor significantly related to attitudes toward food safety practices (as shown in Table 2).

[b] Wholesale business type (n = 1) was excluded from the analysis.

[c] Values in bold were indicated significantly difference with the reference category (*p*-value < 0.05).

Table S4. Multivariable analysis results of consumers.

Final generalized linear models (GLM) and variables [a]	$OR_{adjusted}$	95%CI	*p*-value
**GLM 1: Factors relevant to good knowledge toward COVID-19 (n = 146)**			
**Age group [b]**			
20 – 29	1		
30 – 39	1.03	0.22, 4.86	0.967
40 – 49	2.23	0.42, 11.88	0.347
50 – 60	0.97	0.19, 4.99	0.972
> 60	0.87	0.15, 5.00	0.875
**Education [c]**			
Primary school	1		
Secondary school	**5.92** [e]	**1.39, 25.23**	**0.016**
High school	2.09	0.62, 7.04	0.234
College or higher	**4.74**	**1.25, 17.96**	**0.022**
**Number of family members**			
< 3	1		
3 – 5	**0.34**	**0.13, 0.89**	**0.028**
> 5	**0.10**	**0.02, 0.42**	**0.002**
**GLM 2: Factors relevant to good attitudes toward COVID-19 prevention (n = 142)**			
**Gender**			
Male	1		
Female	**3.09**	**1.03, 9.25**	**0.044**
**Education [c]**			
Primary school	1		
Secondary school	0.91	0.25, 3.28	0.880
High school	**0.32**	**0.10, 1.00**	**0.049**
College or higher	**0.27**	**0.09, 0.83**	**0.022**
**Occupation**			
Private business	1		
Others	**0.13**	**0.03, 0.52**	**0.004**
**Household monthly income (USD) [d]**			
≤ 200	1		
201 – 400	0.74	0.12, 4.51	0.743
401 – 600	0.97	0.15, 6.43	0.978
601 – 800	1.60	0.23, 11.17	0.635
801 – 1,000	6.96	0.80, 60.65	0.079
1,001 – 2,000	1.35	0.13, 13.92	0.803
> 2,000	3.05	0.26, 35.25	0.372
**Number of family members**			
< 3	1		
3 – 5	0.70	0.29, 1.69	0.428
> 5	**0.09**	**0.02, 0.40**	**0.002**
**GLM 3: Factors relevant to good attitudes toward food safety practices (n = 141)**			
**Age group [b]**			
20 – 29	1		
30 – 39	**6.54**	**1.36, 31.55**	**0.019**
40 – 49	3.29	0.76, 14.16	0.110
50 – 60	**11.02**	**2.45, 49.59**	**0.002**
> 60	**5.58**	**1.19, 26.15**	**0.029**
**Occupation**			
Private business	1		
Others	**0.25**	**0.08, 0.82**	**0.022**
**Household monthly income (USD) [d]**			
≤ 200	1		
201 – 400	0.53	0.11, 2.66	0.442
401 – 600	1.18	0.22, 6.32	0.847
601 – 800	1.96	0.31, 12.35	0.472
801 – 1,000	1.06	0.16, 6.90	0.953
1,001 – 2,000	0.47	0.06, 3.74	0.475
> 2,000	2.09	0.18, 24.46	0.558

OR, odds ratio; CI, confidence interval. Value of 1 was the category used as a reference for the comparable category.
[a] Only variables with *p*-value of less than 0.2 were included in the GLM.
[b] Age group of less than 20 (n = 1), [c] illiterate (n = 3), and [d] not available data on household monthly income (n = 5) were excluded from the analysis.
[e] Values in bold were indicated significantly difference with the reference category (*p*-value < 0.05).

Table S4. Multivariable analysis results of consumers (continued).

Final generalized linear models (GLM) and variables [a]	$OR_{adjusted}$	95%CI	*p*-value
**Number of family members**			
< 3	1		
3 – 5	1.04	0.44, 2.50	0.925
> 5	0.37	0.10, 1.37	0.137
**GLM 4: Factors relevant to good practices toward food safety (n = 147)**			
**Education [c]**			
Primary school	1		
Secondary school	**0.27**	**0.09, 0.83**	**0.022**
High school	0.45	0.16, 1.23	0.120
College or higher	0.62	0.25, 1.51	0.291
**Occupation**			
Private business	1		
Others	**0.25**	**0.10, 0.66**	**0.005**

OR, Odds ratio; CI, confidence interval. Value of 1 was the category used as a reference for the comparable category.

[a] Only variables with *p*-value of less than 0.2 were included in the GLM.

[b] Age group of less than 20 (n = 1), [c] illiterate (n = 3), and [d] not available data on household monthly income (n = 5) were excluded from the analysis.

[e] Values in bold were indicated significantly difference with the reference category (*p*-value < 0.05).
